# Delayed diagnosis of X-linked agammaglobulinaemia in a boy with recurrent meningitis

**DOI:** 10.1186/s12883-019-1536-7

**Published:** 2019-12-12

**Authors:** Ya-Ni Zhang, Yuan-Yuan Gao, Si-Da Yang, Bin-Bin Cao, Ke-Lu Zheng, Ping Wei, Lian-Feng Chen, Wen-Xiong Chen

**Affiliations:** Department of Neurology, Guangzhou Women and Children’s Medical Center, Guangzhou Medical University, 9# Jin Sui Road, 510623 Guangzhou City, Guangdong Province People’s Republic of China

**Keywords:** X-linked agammaglobulinemia, Bruton’s tyrosine kinase, Meningitis, Recurrent, Children

## Abstract

**Background:**

X-linked agammaglobulinaemia (XLA) is a rare inherited primary immunodeficiency disease characterized by the B cell developmental defect, caused by mutations in the gene coding for Bruton’s tyrosine kinase (BTK), which may cause serious recurrent infections. The diagnosis of XLA is sometimes challenging because a few number of patients have higher levels of serum immunoglobulins than expected. In this study, we reported an atypical case with recurrent meningitis, delayed diagnosis with XLA by genetic analysis at the second episode of meningitis at the age of 8 years.

**Case report:**

An 8-year-old Chinese boy presented with fever, dizziness and recurrent vomiting for 3 days. The cerebrospinal fluid (CSF) and magnetic resonance imaging (MRI) results were suggestive of bacterial meningoencephalitis, despite the negative gram staining and cultures of the CSF. The patient was treated with broad-spectrum antibiotics and responded well to the treatment. He had history of another episode of acute pneumococci meningitis 4 years before. The respective level of Immunoglobulin G (IgG), Immunoglobulin A (IgA) and Immunoglobulin M (IgM) was 4.85 g/L, 0.93 g/L and 0.1 g/L at 1^st^ episode, whereas 1.9 g/L, 0.27 g/L and 0 g/L at second episode. The B lymphocytes were 0.21 and 0.06% of peripheral blood lymphocytes at first and second episode respectively. Sequencing of the *BTK* coding regions showed that the patient had a point mutation in the intron 14, hemizyous c.1349 + 5G > A, while his mother had a heterozygous mutation. It was a splice site mutation predicted to lead to exon skipping and cause a truncated BTK protein.

**Conclusion:**

Immunity function should be routinely checked in patients with severe intracranial bacterial infection. Absence of B cells even with normal level of serum immunoglobulin suggests the possibility of XLA, although this happens only in rare instances. Mutational analysis of *BTK* gene is crucial for accurate diagnosis to atypical patients with XLA.

## Introduction

X-linked agammaglobulinemia (XLA) is an immunodeficiency disease caused by mutations in the gene coding for BTK, leading to failure to produce mature B lymphocytes [1]. Patients with XLA are subject to recurrent severe bacterial infections from early age and severely reduced B cell and immunoglobulin levels. Most XLA patients have dramatically reduced or absent peripheral blood B cells and of all isotypes of serum immunoglobulins, however, the diagnosis of XLA is sometimes challenging because up to 10–15% of patients have higher levels of serum immunoglobulins than expected [2]. Early diagnosis is important for improving the outcome of XLA. Typical cases of XLA are diagnosed within 5 years of age, however, some cases are identified to have XLA during adolescence, and even in adulthood [3–5]. In this study, we reported an atypical patient with recurrent meningitis, delayed diagnosis with XLA by genetic analysis at the second episode of meningitis at the age of 8 years. Written informed consents were obtained from the parents.

## Case report

An 8-year-old Chinese boy presented with fever, dizziness and recurrent vomiting for 3 days. The patient had been diagnosed with bacterial meningitis in another tertiary hospital and prescribed with cefatriaxone, vancomycin, intravenous immunoglobulin (IVIG) and mannitol for 2 days. He has history of one episode of acute bacterial meningitis at 4 years of age and one episode of pneumonia and sepsis with *Staphylococcus epidermidis* at 5 years old. There was no history of recurrent infections and blood transfusion. He was naturally conceived and born at term by Caesarean section with birth weight being 2.8 kg. He has healthy parents and a healthy 15-year-old female sibling. None of the family’s relatives were known to be subject to recurrent severe infections or were diagnosed with immunodeficiency disorders.

On physical examination, the patient was febrile, tachycardic and tachypneic. Neck rigidity, Kernig’s and Brudzinski’s signs were positive, but there was no focal neurological deficit. Other systemic examinations didn’t reveal any abnormality. His weight was 24.1 kg and height was119.3 cm. His height was at the fifth percentile for age, whereas his weight was within normal range.

**Fig. 1:**
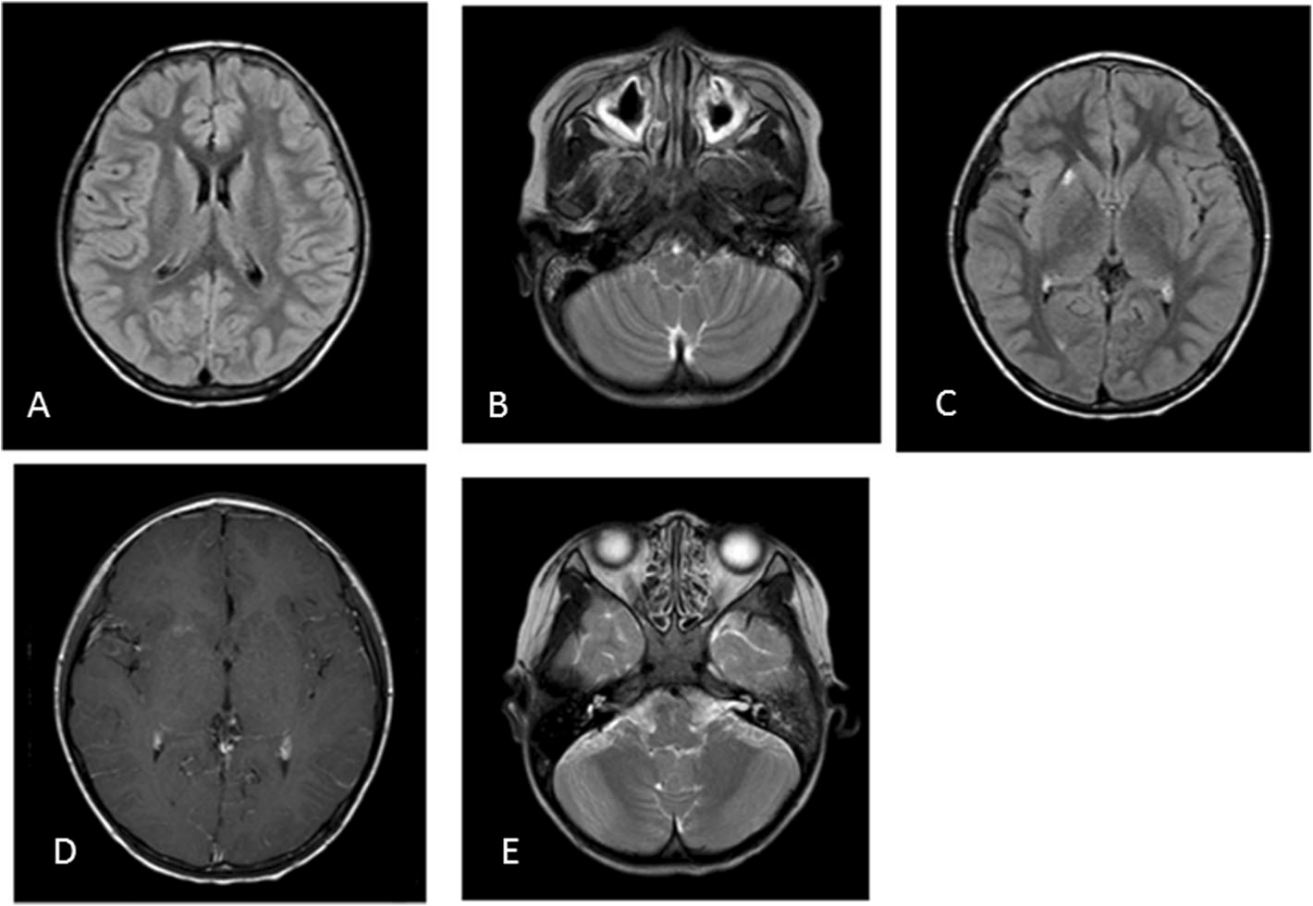
Brain MRI during the two episodes of intracranial infection. A: FLAIR image revealed a little subdural effusion of left frontal, parietal-temporal areas and right frontal area. (1^st^ episode). B: T2-weighted MRI image showedhigh-signal intensity in thickened mucosa of bilateralmaxillary sinus and mastoid process. (1^st^ episode). C: FLAIR image showed high signal on right putamen. (2^nd^ episode). D: Contrast enhanced T1-weighted imagesshowedenhancement of right putamen lesion and bilateral frontal and left temporal lobar meninge. (2^nd^ episode). E: T2-weighted MRI image showedhigh-signal intensity in thickened mucosa of bilateral lethmoidal sinus. (2^nd^ episode)

The level of IgG, IgA and IgM done before IVIG treatment in another tertiary hospital was 1.9 g/L, 0.27 g/L and 0 g/L respectively. A routine blood examination: white blood cell (WBC):40.9 × 10^9^/L,neutrophils:90%,lymphocytes:2%,platelet:322 × 10^9^/L,hemoglobin:102 g/L.C-creative Protein (CRP):243.80 mg/L. Urine and stool analysis was normal. Liver,cardiac and renal function was within normal limits by blood biochemistry examination. Serum lactate and electrolyte were normal. Human Immunodeficiency Virus (HIV) and syphilis serology was negative. Cerebrospinal fluid (CSF) analysis: WBC:548cells/μL, neutrophils:57%, protein:1.83 g/L, glucose:0.96 mmol/L. Gram staining and india ink staining were negative. CSF and blood cultures remained negative. DNA of herpes simplex virus, cytomegalovirus, enterovirus, Epstein-Barr virus, mycoplasma pneumonia and chlamydia pneumonia was negative amplified by polymerase chain reaction (PCR). The immune parameters before treatment in our hospital were shown in Table 1. There was no abnormality in chest X-ray, echocardiography and ultrasound of abdominal and pelvic cavity. Magnetic resonance imaging (MRI) scan of brain suggested meningoencephalitis and nasosinusitis (Fig. 1). The outcome of EEG was normal and brainstem auditory evoked potential (BAEP) showed prolonged latency of bilateral waves I and III respectively. His bone age was normal.

**Table 1:** Immune parameters of two episodes of intracranial infection

Immune parameters	1st episode(4y)	2nd episode(8y)(after IVIG treatment)
Leukocytes (10^9^/L)	22.0↑	40.9↑
Neutrophils (10^9^/L /%)	21.28↑/92	36.81↑/90↑
Lymphocytes (10^9^/L /%)	0.36↓/2↓	0.82↓/2↓
Monocytes (10^9^/L /%)	0.34/2	1.64↑/4
CD45+ cells (cells/μL (normal range))	2116.82 (1661–6643)	931.18 (1661–6643)
CD3+ cells (cells/μL (normal range))/%	2079.84 (805–4459)/98.23↑	857.19 (805–4459)/92.05↑
CD19 + cells (cells/μL (normal range))/%	4.46↓(240–1317)/0.21↓	0.58↓(240–1317)/0.06↓
NK cells (cells/μL (normal range))/%	33.02↓(210–1514)/1.56↓	73.42↓(210–1514)/7.88↓
IgG (g/L (normal range))	4.85↓(5.0–10.6)	7.42 (6.36–14.04)
IgA (g/L (normal range))	0.93 (0.34–1.38)	0.33↓(0.63–1.79)
IgM (g/L (normal range))	0.1↓(0.44–1.44)	0.06↓(0.29–1.41)
IgE (IU/ML (normal range))	49 (0–60)	33 (0–60)
C3 (g/L (normal range))	1.75↑(0.8–1.5)	0.87 (0.8–1.5)
C4 (g/L (normal range))	0.45↑(0.125–0.425)	0.24 (0.125–0.425)

Total T, B, and NK lymphocyte are represented with CD45+ cells, CD3+ cells and CD19 + cells respectively. Values below/above reference ranges are shown with an arrow(↓/↑)

The clinical presentation, neuroimaging findings and results of CSF were suggestive of bacterial meningoencephalitis, despite the negative gram staining and cultures of the CSF. The patient was treated with broad-spectrum antibiotics with vancomycin and meropenem. By the following day, the patient’s fever resolved and dizziness and vomiting disappeared.

During the first episode of bacterial meningitis at 4 years of age, CSF and blood culture showed growth of pneumococci. MRI scan of brain revealed meningitis, subdural effusion, nasosinusitis and mastoiditis (Fig. 1). The immune parameters before treatment were shown in Table 1.The patient received intravenous vancomycin and meropenem and responded well to the treatment.

### Genetic analysis

**Fig. 2:**
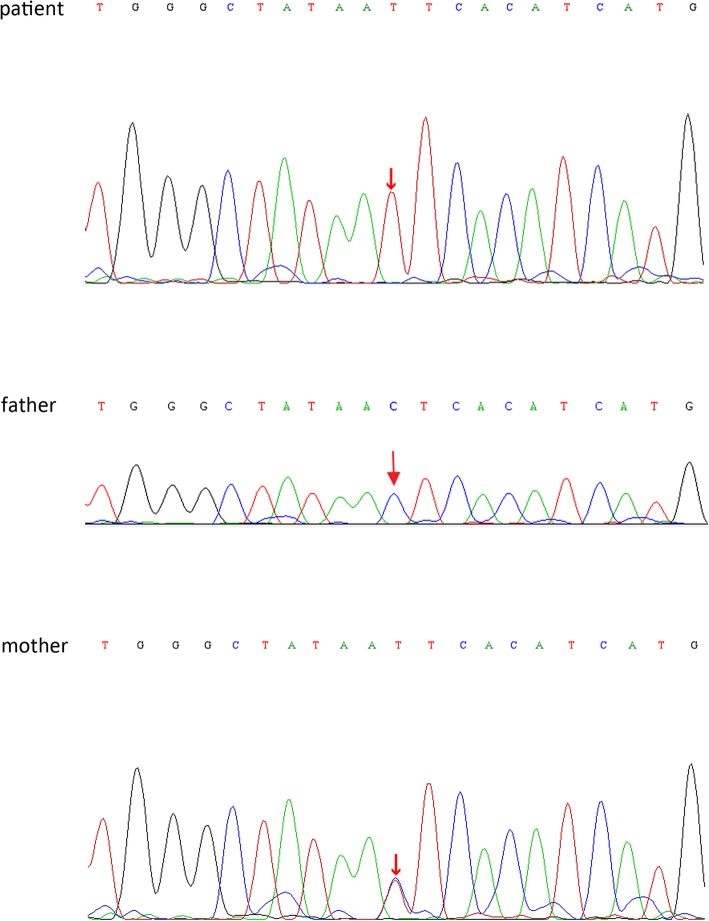
Direct sequence analysis of blood samples from the patient and his parents with reverse sequence

After informed consent had been obtained, genomic DNA was extracted from peripheral blood samples for genetic analysis with immunodeficiencies panel (Mygenostics Inc, Beijing, China). Next generation sequencing was adopted to make genetic analysis, and results were confirmed by Sanger sequence. Sequencing of the *BTK* coding regions revealed a point mutation, hemizygous c.1349 + 5G > A, which is an intron mutation and has been previously reported to be responsible for X-linked agammaglobulinemia [6]. After the diagnosis of proband was confirmed, the *BTK* gene of the parents was analyzed with informed consent signed. Genetic analysis revealed mother had a heterozygous c.1349 + 5G > A, while father was free of any genetic mutations in *BTK*. The results of *BTK* gene sequence analysis with reverse sequence were shown in Fig. 2.

The patient had received IVIG treatments every month since diagnosed with XLA, and had been free from severe infection since then.

## Discussion

According to European Society for Immunodeficiencies (ESID) [2], male patient with less than 2% CD19+ B cells could be diagnosed with probable agammaglobulinemia if all of the following are positive including (1) recurrent bacterial infections in the first 5 years of life;(2) serum IgG, IgM, and IgA more than 2 SD below;(3) absent isohemagglutinins and/or poor response to vaccines;(4) other causes of hypogammaglobulinemia have been excluded. The patient in this study was not diagnosed with XLA until he suffered from second episode of bacterial meningoencephalitis at the age of 8 years. The delayed diagnosis is mainly due to near-normal IgG level and normal IgA level at first episode of bacterial meningitis. Also immunity parameters were not detected during the sepsis at 5 years old. It has been reported that XLA patients carrying *BTK* mutation show normal levels of IgG accompanied with decreased IgM [7].

Plebani et al. performed a survey of 73 Italian XLA patients [3]. Three of the 73 patients had significant levels of serum IgG (approximately 800 mg/dL) at diagnosis. Normal IgA and IgM levels were detected in three and five patients respectively. Only one patient had normal IgA and IgM levels at the same time. During follow-up, serum IgA and IgM decreased to very low levels. In most of the atypical cases, circulating B cells were less than 1%. They suggested that the percentage of circulating B cells, rather than the serum immunoglobulin level, to be a better indicator of XLA. As for our patient, he had near-normal level of serum IgG and normal IgA at 4 years old. Four years after, the serum IgG and IgA declined significantly, consistent with the outcomes of the cases reported by Plebani [3]. The mechanism of the immunoglobulin level decreasing with time is not clear, other genetic or epigenetic factors may influence the immunological phenotype of XLA. Basile et al. reported that absence of tonsils occurred in almost 80% of XLA patients, and suggested absent tonsils and the absence of B cells to be the main clues for early diagnosis [7]. As for our patient, B lymphocytes were also below 2% of peripheral blood lymphocytes at the both episodes of meningitis, while the tonsil was present.

The *BTK* gene is located at Xq21.3-Xq22 and contained 19 exons. The BTK protein has five different functional domains including PH, TH, SH3, SH2, and SH1 [8]. ALL the 5 domains are important for sustaining the normal function of BTK [9]. The activity of the tyrosine kinase and the maturation of pre-B cells can be influenced by mutations in any of these five domains [10, 11]. Mutations of the *BTK* gene can occur in the exons, introns, and promoters [10, 11]. Missense mutations dominate the mutations, followed by nonsense mutations, splice site mutations, insertions, and deletions. The mutation c.1349 + 5G > A in the present patient is a splice site mutation located in the intron 14, which has been reported before and been predicted to reduce the stability of base-pairing of the 5′ splice site with the 5′ end of Ul small-nuclear ribonucleic acid (RNA) [6]. This mutation is speculated to affect the SH1 domain [6], which is the catalytic domain for Tyr phosphorylation [9]. Carrillo-Tapia et al. [4] had summarized the data of atypical XLA from several studies and found 28% of total 37 patients had missense changes in the SH1 domain.

The previously reported [6] patient with c.1349 + 5G > A mutation had typical XLA (total absence of B cells, IgA 0.08 g/L, IgG 0.22 g/L and IgM 0.25 g/L at diagnosis) with no history of growth hormone deficiency. However, our patient has atypical XLA with low level of B cells and IgM but near-normal level of IgG and normal IgA during the first episode of meningitis. Clinical heterogeneity has been reported in a number of studies. It was reported that an XLA patient with missense mutations in the PH domain presented with episodes of severe primary infections, but whose brother with the same mutation remained healthy [12]. It is proposed that other genetic and environmental factors might affect the diverse phenotypes of XLA. Another case report showed a G to A transition in position + 5 of 5′ splice site of *BTK* intron17 in a patient with XLA associated with growth hormone deficiency [13]. The present patient had short stature but normal bone age. However, no further test was performed for growth hormone deficiency. Flow cytometry or western blot should be performed in the future to detect the BTK expression and verify the functional effect of the mutation c.1349 + 5G > A.

## Conclusion

Early diagnosis of XLA is crucial, because the affected patients are subject to recurrent and severe infections unless they receive IVIG replacement therapy. Immunity function should be routinely checked in patients with severe infection including intracranial bacterial infection. Absence of B cells even with normal level of serum immunoglobulin should alert physicians to further confirm the possibility of XLA. Mutational analysis of *BTK* gene is important for accurate diagnosis to atypical patients with XLA.

## Data Availability

The datasets used in the current study are available from the corresponding author on reasonable request.

## Abbreviations

BAEP: Brainstem Auditory Evoked Potential
BTK: Bruton’s tyrosine kinase
CRP: C-reactive protein
CSF: Cerebrospinal fluid
EEG: Electroencephalography
FLAIR: Flow attenuated inversion recovery
HIV: Human immunodeficiency virus
IVIG: Intravenous immunoglobulin
MRI: Magnetic resonance imaging
TB: Tuberculosis
WBC: White blood cell
XLA: X-linked agammaglobulinemia
